# Volatile Profiling of Aromatic Traditional Medicinal Plant, *Polygonum minus* in Different Tissues and Its Biological Activities

**DOI:** 10.3390/molecules191119220

**Published:** 2014-11-20

**Authors:** Rafidah Ahmad, Syarul Nataqain Baharum, Hamidun Bunawan, Minki Lee, Normah Mohd Noor, Emelda Roseleena Rohani, Norashikin Ilias, Noraziah Mohamad Zin

**Affiliations:** 1 Metabolomics Research Laboratory, Institute of Systems Biology, Universiti Kebangsaan Malaysia, Bangi 43600, Selangor, Malaysia; E-Mails: fida.ahmad@ukm.edu.my (R.A.); hamidunb@yahoo.com (H.B.); normah@ukm.edu.my (N.M.N.); emelda@ukm.edu.my (E.R.R.), ekin86@ukm.edu.my (N.I.); 2 Department of Bio-Environment Chemistry, Chungnam National University, 99 Daehak-Ro, Yuseong-Gu, Daejon 305-764, Korea; E-Mail: mklove22@nate.com; 3 Programme of Biomedical Science, Faculty of Health Sciences, School of Diagnostic and Applied Health Sciences, Universiti Kebangsaan Malaysia, Kuala Lumpur 50300, Malaysia; E-Mail: noraziah.zin@ukm.edu.my

**Keywords:** *Polygonum minus*, volatile compound, different tissues, solid-phase microextractions, gas chromatography, biological activities, essential oil

## Abstract

The aim of this research was to identify the volatile metabolites produced in different organs (leaves, stem and roots) of *Polygonum minus*, an important essential oil producing crop in Malaysia. Two methods of extraction have been applied: Solid Phase Microextraction (SPME) and hydrodistillation coupled with Gas Chromatography-Mass Spectrometry (GC-MS). Approximately, 77 metabolites have been identified and aliphatic compounds contribute significantly towards the aroma and flavour of this plant. Two main aliphatic compounds: decanal and dodecanal were found to be the major contributor. Terpenoid metabolites were identified abundantly in leaves but not in the stem and root of this plant. Further studies on antioxidant, total phenolic content, anticholinesterase and antimicrobial activities were determined in the essential oil and five different extracts. The plant showed the highest DPPH radical scavenging activity in polar (ethanol) extract for all the tissues tested. For anti-acetylcholinesterase activity, leaf in aqueous extract and methanol extract showed the best acetylcholinesterase inhibitory activities. However, in microbial activity, the non-polar extracts (n-hexane) showed high antimicrobial activity against Methicillin-resistant *Staphylococcus aureus* (MRSA) compared to polar extracts. This study could provide the first step in the phytochemical profiles of volatile compounds and explore the additional value of pharmacology properties of this essential oil producing crop *Polygonum minus*.

## 1. Introduction

*Polygonum minus* (Polygonaceae) is an important aromatic plant in Malaysia and is widely used in South East Asia, especially in Thailand, Laos, Indonesia and Vietnam, as a flavouring ingredient in food and folk medicines. The Malaysian Government has listed this plant in the National Agro-Food Policy to ensure sufficient supply of this food and to strengthen the agricultural economy. *P. minus* has been recognized by the Malaysian government in the Herbal Product Blueprint as an essential oil producing crop [1]. *P. minus* oil is a high potential source of natural aliphatic aldehydes and can be produced economically in North East Victoria, Australia [2]. In Japan, China, and Europe, *P. minus* has long been used as a hot-tasting spice. Traditionally, *P. minus* has been used to treat digestive disorders, to reduce dandruff and as a treatment for poor eyesight [3]. Recent studies showed that this plant demonstrated anti-ulcer, anti-inflammatory [4], anti-ageing [5], antioxidants and immunomodulatory activity [6]. In India, *Polygonum* sp., has been used traditionally to treat diuretic, CNS stimulant, diaphoretic, stomachic, styptic, in bleeding and in diarrhea [7]. Other species of the Polygonaceae family have been reported for their effectiveness in cerebral ischemia [8], Parkinson’s disease [9] and neuroprotective effects [10]. The volatile compounds in this medicinal plant are recognized as an important part for its pharmacological activities mentioned above. Recently, *P. minus* has been characterized by their functional group of metabolites through Fourier-Transform Infrared Spectroscopy (FTIR) analysis under different temperature treatment [11] and Liquid Chromatography Mass Spectrometry (LCMS) profiling has been conducted for the production of flavonoid [12]. A previous study by Baharum *et al.* [13], and Yaacob [14] described the composition of the essential oil in *P. minus* leaves. However, the chemical composition of *P. minus* from other tissues has never been reported. Roots of many medicinal plants are often used in drugs constituents due to its capability of accumulating a high level of medicinal related secondary metabolites [15]. Some studies have successfully identified valuable compounds from *Polygonum sp.* roots. For example, researchers have found phytoestrogens in *P. cuspidatum* [16], phytohormones and anthraquinones in *P. multiflorum* roots [17] and indigo in *P. tinctorium* roots [18]. Thus, understanding the metabolites found in different tissues could relate further with its biological potential. 

In this study, our aim was to identify and profile the volatiles metabolites in different organs of *Polygonum minus*, an important crop for flavour and food industry in Malaysia using SPME and hydrodistillation technique coupled with Gas Chromatography-Mass Spectrometry (GC-MS). SPME is a powerful technique, a solvent-free method that reduces chemical changes in analytes and artefact formation for the analysis of the volatile constituents [19,20]. Hydrodistillation technique has been used widely in the essential oil industry despite the need for chemical alterations and the heat sensitive compounds are easily destroyed. To discriminate between samples, the output (the percentage area of the peak) will be interpreted in principal component analysis (PCA). Further study to evaluate the additional value of essential oil in comparison to solvent extracts has been highlighted. Determination of antioxidant properties, evaluation of anticholinesterase activity and screening of antimicrobial assay will be conducted to highlight the potential biological activities in essential oil in this aromatic plant. 

## 2. Results and Discussion

### 2.1. Volatile Profiles of Different Tissues from SPME Extraction

**Figure 1:**
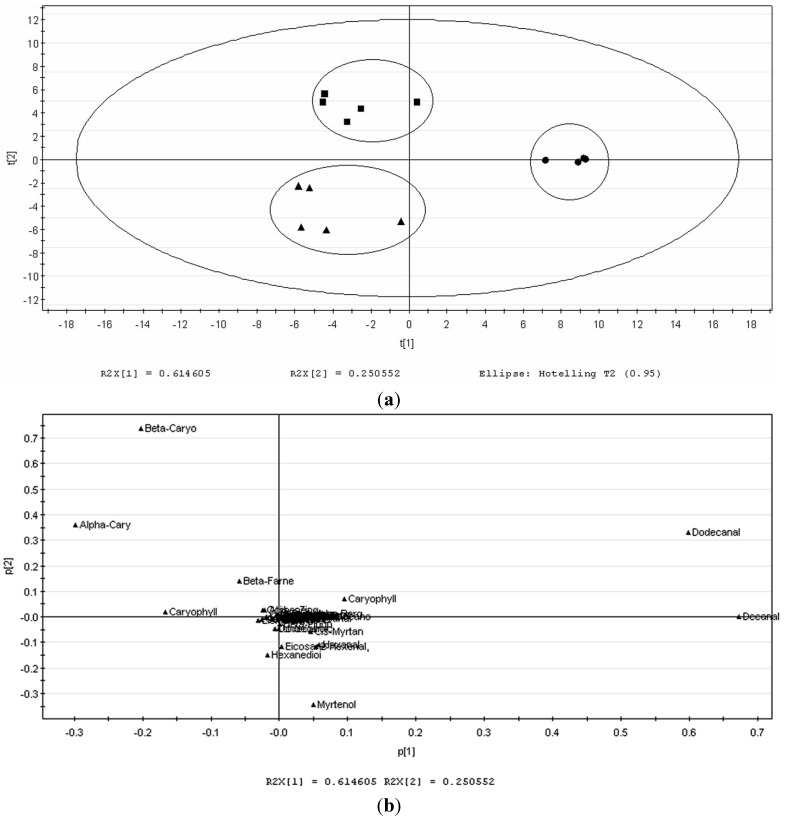
Score plot of (**a**) and PCA loading (**b**) of volatile compounds in *P. minus* in different tissues by GC-MS. Leaf (●), stem (■) and root (▲) using SPME.

SPME extracts of the volatile compounds present in the leaves, stems and roots were identified by GC-MS. PCA from data matrix was then performed in each one of the different groups in order to find the main contributions of variability and to establish the relation between varieties and volatile compounds. The volatile profiles of the different tissues are shown in Figure 1a with clear separation between parts of plant. The result of PCAs provided a plot of the principal component (PC) scores for the most important PCs (PC1 *vs*. PC2). The first two PCs account for 86.51% of the total variation (PC1: 61.4% and PC2: 25.1%). The R2 and Q2 values are 0.975 and 0.709, respectively. The stems and roots were differentiated from the leaves by PC1 (61.4%), whereas the stems were separated from the roots across PC2 by 25.1%. Figure 1b shows the corresponding loadings plot that establishes the relative importance of each variable. β-caryophyllene, dodecanal and decanal were found as the unique compounds that scattered far apart from the cluster that contributed mainly to the separation of volatiles in different tissues. The variables that influenced PC1 value are dodecanal (0.6) and decanal (0.68). On the other hand, in the case of PC2 value, β-caryophyllene (0.75) seems to contribute more compared to other compounds on the loading plot (Figure 1b).

Most of the volatiles detected in both the leaves and stems were sesquiterpenes, followed by aliphatic and monoterpene compounds as shown in Table 1. The most abundant sesquiterpene in the leaves, stems and roots was β-caryophyllene, at 5.78%, 34.71% and 22.92%, respectively. β*-*caryophyllene was also one of the major compounds detected in *Polygonum hydropiper*, a closely related species in *P. minus* [21]. The most abundant monoterpene in the leaves and stems was α-pinene and the most abundant monoterpene in the roots was (*Z*)-myrtanol which can only be detected in roots. The relative concentrations of several classes of volatile compounds in *P. minus* are shown in Table 2. Although the number of terpenes were abundantly found in leaves, the relative concentrations of terpenes were found highly in stems (55.35%), followed by roots (28.07%) and leaves (10.59%). This was due to the very high relative concentration of aliphatic compounds (decanal: 41.56%) and (dodecanal: 45.93%) in the leaves compared to the stems and roots. In contrast, more than 50% of the relative compound concentrations in the roots were due to organic acids (Table 1 and Table 2). 

Most of the terpenoids detected by GC-MS were abundantly present in the leaves compared to the stems and roots. Aharoni *et al.* [22] showed that volatile compounds, such as terpenoids, were emitted from aerial parts of the plant. The distinct distribution of terpenoids between leaves, stems and roots was in line with the current knowledge of terpenoids, based on a few environmentally related factors. The lack of terpenoid compounds is a predictable result, given that roots are not exposed to oxidative stress in the same way that leaves and stems are and do not have a photosynthetic function. According to Opitz [23], a large variety of terpenes are produced constitutively in all photosynthetically active parts of the plant and stored in the subepidermal glands. Temperature also plays a role in terpenoid production in plants, especially the aerial parts, which are more exposed to high temperatures. Temperature increases the production and emission rates of most terpenes exponentially, up to a maximum limit, by enhancing enzyme synthesis activity, raising the terpene vapor pressure and decreasing the resistance of the emission pathway [24,25,26].

**Table 1:** Volatile profiling of leaves, stems and roots of *P. minus* by SPME technique and essential oil by using gas chromatography mass spectrometry.

No	Compound ^a^	Retention Indices ^e^	SPME Technique			Essential Oil		
			Percentage ^b^					
			Leaves	Stems	Roots	Leaves	Stems	Roots
	*Terpenes(Monoterpenes)*							
1	α-Pinene	939	0.52 ^c^	0.25	-^d^	0.256	0.152	2.122
2	α-Thujene	938	0.011	-	-	-	-	-
3	Limonene	1030	0.022	0.084	-	-	-	-
4	β-Ocimene	1038	0.022	0.388	-	-	-	-
5	Myrtenal	1233	-	0.216	0.496	-	-	-
6	-(Z)-Myrtanol	1288	-	-	0.602	-	-	-
7	Borneol	1162	0.081	0.095	-	-	-	-
8	(*E*)-Geranyl acetone	1448	-	-	0.313	-	-	-
	*Terpenes (sesquiterpenes)*							
9	Germacrene D	1484	0.093	-	-	-	-	-
10	Isocaryophyllene	1438	0.012	-	-	0.296		-
11	Copaene	1377	-	0.359	-	-	-	-
12	α-Zingiberene	1494	0.144	0.186	-	0.046	-	-
13	δ-Elemene	1335	-	1.234	-	-	-	-
14	Aromadendrene	1475	0.159	-	-	0.3816	-	-
15	(*E*)-α-Bergamotene	1431	1.383	-	1.855	0.984	1.339	0.666
16	β-Guaiene	1483	-	-	-	0.068	-	-
17	4,11-selinadiene	1474	0.024	-	-	0.181	0.055	0
18	β-Caryophyllene	1467	6.063	34.71	22.99	12.888	19.564	8.851
19	Eremophilene	1503	0.353	-	-	-	-	-
20	β-farnesene	1445	0.527	5.002	0.803	0.1539	-	-
21	Sesquiphellandrene	1560	0.102	0.13	-	0.053	0.19	-
22	Alloaromadendrene	1496	0.029	-	-	-	-	-
23	α-Bisabolene	1506	0.131	-	-	-	-	-
24	α-Panasinsen	1381	0.366	-	-	0.475	1.807	0.455
25	α-Cedrene	1410	0.79	-	-	-	-	-
26	Valencene	1490	-	2.114	-	-	1.005	-
27	Nerolidol	1539	0.084	-	-	0.246	0.336	-
28	α-Himachalene	1449	-	1.276	-	0.513	0.249	-
29	Cadinene	1543	-	0.329	-	-	0.301	-
30	Gurjunene	1412	-	2.067	-	-	-	-
31	Caryophyllene oxide	1573	0.194	4.883	0.992	0.683	4.107	-
32	Humulene epoxide	1642	-	2.023	-	-	3.225	-
33	Seychellene	1444	-	-	-	0.481	1.429	-
34	α-Curcumene	1553	-	-	-	0.185	0.084	-
	*Terpenes (sesquiterpenes)*							
35	Cubenol	1645	-	-	-	-	0.653	-
36	Thujopsene	1429	-	-	-	0.13	-	-
37	Longipinocarvone	1398	-	-	- ^d^	0.492 ^c^	0.7824	-
38	Aristolene	1449	-	-	-	0.109	-	-
39	Cyclolongifolene oxide, dehydro-	1657	-	-	-	1.71	-	-
40	α-Cadinol	1676	-	-	-	-	0.75	-
41	β-Bisobolol	1666	-	-	-	0.373	2.449	-
42	α-Eudesmol	1896	-	-	-	-	5.416	-
43	Drimenol	1766	-	-	-	2.219	0.286	-
44	Isolongifolol	1716	-	-	-	3.323	-	-
45	Drimenin	1941	-	-	-	0.287	-	-
	*Aliphatic compounds*							
46	Undecane	1101	0.194	0.209	-	-	-	-
47	Decanal	1209	41.563	4.915	1.181	11.629	10.308	10.48
48	Decane, 4-methyl	1059	0.039	-	-	-	-	-
49	Decanol	1274	-	0.548	-	-	0.463	-
50	Undecanal	1308	0.424	0.163	-	0.151	0.078	-
51	Cyclohexane, 1-ethenyl-1-methyl-2,4-bis(1-methylethene)	1392	0.134	-	0.577	-	-	-
52	Dodecanal	1413	45.927	37.45	10.92	59.457	42.121	24.501
53	Pentadecane	1500	0.445	-	-	-	-
54	1-Dodecanol	1469	-	0.228	-	-	-	-
55	Pentadecanal	1711	0.559	-	-	1.687	-	-
56	Heptadecane	1700	-	0.093	-	-	-	-
57	Octadecane	1800	-	0.165	1.572	-	-	-
58	1-Hexadecanol	1870	-	0.188	0.59	-	-	-
59	Eicosane	2000	-	0.165	1.572	-	-	10.568
60	Tetracosane	2400	-	-	0.59	-	-	2.271
61	Bicyclo[5.3.0]decane, 2-methylene-5-(1-methylvinyl)-8-methyl-	1456	-	-	-	0.017	-	-
62	Bicyclo[5.2.0]nonane, 2-methylene-4,8,8-trimethyl-4-vinyl-	1458	-	-	-	0.096	-	-
63	Naphthalene, 1,2,3,4,4a,5,6,8a-octahydro-4a,8-dimethyl-2-(1-methylethenyl)-,	1498	-	-	-	-	0.286	-
64	Phytane	1809	-	-	-	-	0	2.158
	*Aliphatic compounds*							
65	Perhydrofarnesyl acetone	1913	-	-	-	-	0	0.953
66	3,7,11,15-Tetramethyl-2-hexadecen-1-ol	1942	-	-	-	-	0.404	-
67	Phytol	2128	-	-	- ^d^	0.365 ^c^	0.143	-
	*Organic acids*							
68	Dodecanoic acid	1564	-	0.193	1.277	-	-	4.568
69	Myristoleic acid	1719	-	-	0.924	-	-	-
70	Tetradecanoic acid	1720	-	-	8.926	-	-	-
71	Pentadecanoic acid	1851	-	-	5.17	-	-	1.485
72	Hexadecanoic acid	1959	-	-	18.15	-	-	23.471
73	Oleic acid	2141	-	-	2.388	-	-	-
74	Octadecanoic acid	2124	-	-	1.42	-	-	-
75	Hexanedoic acid	1871	-	0.337	16.692	-	-	-
76	2-Propenoic acid	1380	-	-	-	-	-	2.023
77	1,2-Benzenedicarboxylic acid	1915	-	-	-	-	-	2.902

^a^ As identified by GC-MS Software; names according to NIST mass spectral library; ^b^ Percentage of each component is calculated as peak area of analyte divided by peak area of total ion chromatogram times 100; ^c^ The results are the mean at least 3 experiments; ^d^ Not detected or percentage of the component is lower than 0.01%; ^e^ The retention indices based on alkane series.

**Table 2:** Relative concentrations of several classes of compound in *P. minus.*

Chemical Class of Compound	SPME Technique			Essential Oil		
	% Relative Area			% Relative Area		
	Leaves	Stems	Roots	Leaves	Stems	Roots
Terpenes						
Monoterpenes	0.136	1.033	1.41	0.256	0.152	2.122
Sesquiterpenes	10.926	54.314	26.637	26.279	44.027	9.973
Total Terpenes	10.59	55.347	28.051	26.535	44.179	12.094
Aliphatic compounds	88.069	44.121	17.007	73.402	53.803	50.931
Organic acids	0	0.530821	54.95	0	0	34.449

Apart from high terpenoid production in the aerial parts of *P. minus*, this study also found high levels of decanal and dodecanal, the two main aldehydes that contribute to the flavor of *P. minus.* The highest levels for these compounds were found in the leaves (41.56% and 45.93%, respectively) and stems (4.92% and 34.75%, respectively), but were much lower in the roots (1.18% and 10.92%, respectively) (Table 1). Similar findings for these two main aldehydes were observed in the essential oils produced by *P. minus* leaves by Baharum *et al.* [13] and Yaacob [14]. This indicated that both compounds could constitute a good phytochemical marker in quality control to distinguish between powdered leaves, stems and roots. In *P. hydropiper*, the stems appeared to contain more decanal and dodecanal, but less esters than the leaves [21]. The high levels of aldehydes, such as dodecanal and decanal, in the leaves and stems may be due to enhanced UV-B irradiation exposure [27]. Enhanced UV-B radiation may also have increased isoprene and terpenoid production in the plants [28,29,30]. 

This study had also detected eight organic acid compounds in roots (Table 1). The most abundant organic acid in the roots was hexadecanoic acid (18.15%). The composition of organic acids that accumulate varies depending upon species, age of the plant and tissue type. The high accumulation of organic acids in plant tissues is most probably due to their important role as photosynthetic intermediates. Organic acids can be either accumulate in the vacuole or be excreted into the apoplast by specific carrier proteins that are transported towards the phloem and then directed to the roots for exudation [31]. It has also been well documented that plant roots exude a variety of organic compounds in response to: soil stress, for example, mineral nutrient deficiency, aluminium tolerance and plant–microbe interactions [32]. Further analysis of organic acid compounds in different tissues should be carried out in order to support these findings using more suitable analytical tools, such as LC-MS TOF.

### 2.2. Volatile Profiles of Different Tissues from Essential Oils

**Figure 2:**
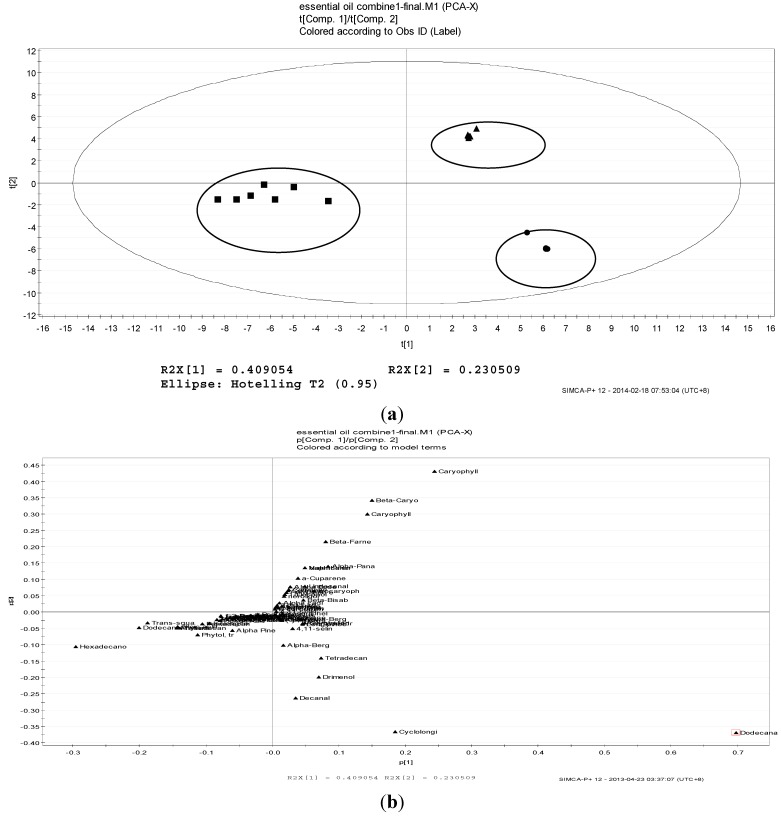
Score plot of (**a**) and PCA loading (**b**) of essential oil in *P. minus* in different tissues. Leaf (●), stem (■) and root (▲).

The volatile profiles of essential oil of the different tissues are shown in Figure 2a. The positioning of metabolic patterns, from left to right, increases understanding of the volatile constituents in the different tissues. The first two PCs account for 63.96% of the total variation (PC1: 40.91% and PC2: 23.05%). The R2 and Q2 values are 0.757 and 0.48, respectively. The stems and leaves were differentiated from the roots by PC1 (40.91%), whereas the stems were separated from the leaves across PC2 by 23.05%. As shown in Figure 2b, dodecanal were the unique compounds which scattered far apart from the cluster that contributed mainly to the separation of volatiles in different tissues. The variable which influenced the PC1 value is dodecanal (0.75) whereas the variables which influenced the PC2 value are carryophyllene oxide (0.44) and cyclolongifolene oxide, dehydro (−0.36).

From PCA analysis, which shows separation of volatiles from different tissues, we have detailed out the results to identify potential compounds in each sample in Table 1. Table 2 shows several compounds, belonging to distinct chemical classes, including: terpenes (monoterpenes and sesquiterpenes), aliphatic compounds and organic acid compounds and the percentage yield of the essential oil in different tissues. The obtained essential oil had a yellowish colour. A total of 31 compounds was identified in the leaf oil, 27 in the stem oil and 15 in the root oil with dodecanal and decanal being the most dominant one in all the tissues especially leaves (59.46% and 11.63%), stems (42.12% and 10.31%) and roots (24.50% and 10.48%). 

In Table 2, most of the chemical composition detected in leaf, stem and root comprised aliphatic compounds (73.10%, 53.88% and 50.93%, respectively), meanwhile stems have the highest relative concentrations of terpenes (44.18%) compared to leaves (26.54%). Roots have the highest concentration of organic acid (34.43%), however the terpenes concentration of root is the lowest compared to the other tissues (12.09%). Organic acid was not detected in leaves and stems.

**Figure 3:**
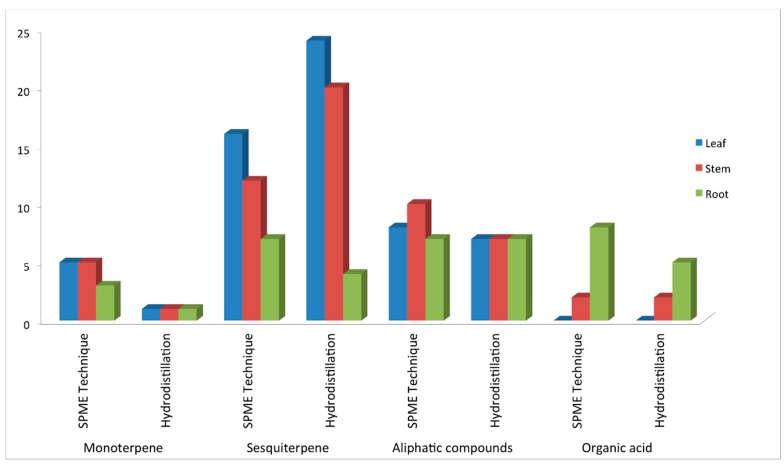
Number of compounds found by the SPME technique and the hydrodistillation technique.

The number of compounds found by using the SPME technique and hydrodistillation technique in all tissues are shown in Figure 3. There were more compounds found in *P. minus* with use of the SPME technique especially in monoterpene group, aliphatics group and organic acid group compared to hydrodistillation technique. This might be due to the power and sensitivity of SPME technique. 

### 2.3. Antioxidant Activity, Total Phenolic Content and FRAP

The potential antioxidant activity of the extracts and the essential oil was determined on the basis of scavenging activity of the stable free radical DPPH. The highest antioxidant activity is in polar extracts (methanol, ethanol and aqueous). In Table 3, ethanol extract showed the highest antioxidant activity for all the tissues (leaf, stem and root) with IC_50_ of 31.864 ± 1.0340 µg/mL, 30.430 ± 1.0281 µg/mL, 63.577 ± 1.0281 µg/mL, respectively, followed by methanol extraction for all tissues (leaf, stem and root) with IC_50_ of 38.309 ± 1.245 µg/mL, 37.154 ± 1.0628 µg/mL and 93.325 ± 1.0407 µg/mL, respectively. In both extracts, stem showed the highest antioxidant activity, however there is no significant difference between leaf and stem in the same extracts. Non-polar extracts (DCM and hexane) did not exhibit a good antioxidant activity; in addition, the IC_50_ value could not be determined in the extract. Due to low antioxidant activity, we did not carry out experiment on the total phenolic content and FRAP analysis in non-polar extract (n-hexane and dichloromethane). The essential oil from different tissues has also been tested of DPPH radical scavenging activity. The DPPH scavenging activity of leaf and stem from essential oil, demonstrating IC_50_ value of 3388 ± 1.085 µg/mL and 4570 ± 0.089 µg/mL, respectively, but no activity was found in root.

Methanol extract has the highest total phenolic content in leaf and stem with 241.19 mg·GA/g and 239.48 mg·GA/g, respectively (Table 3). Root has the lowest total phenolic content in all the polar extracts. This correlates well with the low DPPH scavenging activity in root extract. Meanwhile, leaf shows the highest total phenolic content in all the polar extracts. Typical phenolics that possess antioxidant activity are mainly phenolics acid and flavonoid. Although total phenolic contents are higher in methanol extracts compared to ethanol extracts, the DPPH scavenging activity of ethanol extracts are higher than methanol extract. According to Katsube *et. al* [33], total phenolic content measured by Folin-Ciocalteu does not give a full picture of the quality and quantity of the phenolic constituents in the extracts. For FRAP analysis, ethanol extracts have the highest FRAP value which correlates well with DPPH scavenging activity. The FRAP value and the DPPH scavenging activity showed the same trend where a positive correlation exist between the antioxidant capacity and the reducing capability of the extract. This is due to the same mechanism which involved capability of the reducing radicals (ferric ion and DPPH free radicals) in the assay. According to Hassim *et al.* [34], the best antioxidant activity in *P. minus* was achieved on 70% methanol extract followed by ethanol and aqueous extracts using supercritical fluid extraction (SFE). However, we have improved the extraction method by using 100% solvent for better antioxidant results. Overall results showed that leaf and stem have the highest antioxidant activity which might be due to the high number of terpenes found in leaf and stem compared to root (Figure 3). The similar trends were reported by Di Vaio *et al.* [35] which showed that the antioxidant activity from lemon cultivars was linked mainly to terpene components although a slight contribution is made by other substances with radical scavenger properties present in the extract. On comparing the results for antioxidant activity with those of essential oil, there emerged little correlation between essential oil content and antioxidant activity of the solvent extracts. This could be due to the occurrence in solvent extracts of other phytochemical compounds (that constitute the non-volatile fraction of essential oils such as cumarins, psoralens and phenols) that have antioxidant activity and that are not detected by GC-MS analysis.

The contribution of the organic acids content to the antioxidant activity was considered. However, among compounds reported to have stronger antioxidant activity, (ascorbic acid and citric acids), none was found in roots although roots have an abundance of organic acids.

**Table 3:** DPPH radical scavenging activity, Total Phenolic Content and FRAP of extracts.

Solvent or Standard	Plant Tissue	DPPH (µg/mL) (IC 50)	Total Phenolic Content (mg GA/g of Extract)	FRAP Value (µmol Fe (II)/g Extract)
Methanol	Leaf	38.309 ± 1.2450 * ^g^	241.19	77.40
	Stem	37.154 ± 1.0628 ^g^	239.48	50.87
	root	93.325 ± 1.0407 ^de^	66.21	21.28
Ethanol	Leaf	31.864 ± 1.0340 ^f^	131.11	92.19
	Stem	30.430 ± 1.0281 ^g^	115.26	52.91
	root	63.577 ± 1.0281 ^g^	100.03	65.66
Aqueous	Leaf	74.131 ± 1.0471 ^ef^	154.65	55.97
	Stem	99.243 ± 1.0281 ^d^	112.82	33.01
	root	-	11.5	1.89
Dichloromethane	Leaf	-	n.d	n.d
	Stem	-		
	Root	-		
n-hexane	Leaf	-	n.d	n.d
	Stem	-		
	Root	-		
Essential Oil	Leaf	3388 ± 1.085 ^k^	n.d	n.d
	Stem	4570 ± 0.089^ l^		
	Root	n.d		
Gallic Acid		3.3627 ± 1.034 ^i^	n.d	n.d
Ascorbic Acid		22.73526 ± 1.114 ^h^	n.d	n.d

Means in a column followed by a different lowercase letter represents results are significantly different (ANOVA *p* < 0.05, Tukey test); * Standard error; n.d—not determined.

### 2.4. Acetylcholinesterase Activity (AchE)

Acetylcholinesterase activity (AchE) is found among neurofibrillary and neuritic plaques and its inhibition is an effective tool for the treatment of Alzhemier’s disease and related dementia. All known acetylcholinesterase inhibiting drugs, for example, tacrine used in Alzemier’s disease have adverse effects such as hepatotoxicity and gastrointestinal [36]. Therefore, research is focusing on the development of new acetylcholinesterase inhibitors with less toxicity from plant extracts [37]. Nevertheless, there are no toxicity effects againts normal human lung cells when tested with leaves of *P. minus* extract up to 500 µg/mL concentrations [38]. Due to remarkable results of antioxidant activity, we carried out this study as it is suggested that antioxidant activity has a significant role in the treatment of Alzheimer’s disease with relation to the acetylcholinesterase enzyme [39]. Moreover, other *Polygonum sp*. have been used traditionally as Central Nervous System (CNS) stimulant [7]. 

In this study, we tested all parts of plant extracts and essential oil (leaves, stems and roots) of *P. minus* of acetylcholinesterase inhibition. No activity was found in essential oil up to a concentration of 10 mg/mL (Table 4). However, leaf in aqueous extracts and methanol extracts showed the best acetylcholinesterase inhibitory activities, demonstrating IC_50_ value of 234 ± 0.0087 µg/mL and 342.768 ± 1.0568 µg/mL, respectively. Stem extract did not exhibit a good activity for acetylcholinesterase inhibitors except for DCM extract and aqueous extract demonstrating IC_50_ value of 478 ± 0.0174 µg/mL and 581 ± 0.036 µg/mL. Root has the lowest anticholinesterase activity and some of the extract did not show any activity. Only methanol and DCM extracts of root showed anticholinesterase activity at IC_25_ value of 1005.77 ± 1.776 µg/mL and 1800 ± 0.081 µg/mL, respectively. We could not calculate the IC_50_ value because of its low activity. Overall, the non-polar extracts (DCM and haxane) did not show a good anticholinesterase activity compared to polar extract (aqueous, methanol and ethanol). The most probable reason for their potential acetylcholinesterase activity might be related to the presence of terpenoids. These terpenoids, on the other hand, due to their small molecular size and lipophilicity, readily cross the blood-brain barrier and are effective in the treatment of Alzheimer diseases [40]. A previous study of anticholinesterase activity of *Polygonum hydropiper* [41] showed that the crude extract of methanol has an IC_50_ value of 330 µg/mL with starting material of 4.5 kg and this plant is considered to have potential use in neurodegenerative treatment. When compared to these results, we believe that *P. minus* has more potential to become an Alzheimer’s treatment.

**Table 4:** Anticholinesterase activity in different extracts.

Solvent or Standard	Plant Tissue	Anticholinesterase Activities (IC50)
Methanol	Leaf	342.768 ± 1.0568 *^a^
	Stem	809.096 ± 1.1003 ^b^
	Root	1005.77 ± 1.776 ^bi^ (IC 25)
Ethanol	Leaf	910 ± 0.0123 ^c^
	Stem	930 ± 0.0071 ^c^
	Root	-
Aqueous	Leaf	234 ± 0.0087 ^d^
	Stem	581 ± 0.036 ^ce^
	Root	-
Dichloromethane	Leaf	770 ± 0.0187 ^f^
	Stem	478 ± 0.0174 ^cg^
	Root	1800 ± 0.081 ^hi^ (IC 25)
n-hexane	Leaf	-
	Stem	-
	Root	n.d
Essential Oil	Leaf	-
	Stem	-
	Root	-
Tacrine		2.59 ± 0.012 ^j^

Means in a column followed by a different lowercase letter represents results are significantly different (ANOVA *p* < 0.05, Tukey test); * Standard error. n.d—not determined.

### 2.5. Antibacterial Activity

*P. minus* leaves, were extracted out using five different solvents; hexane, dichloromethane (DCM) methanol, ethanol and aqueous. However, only three solvents were chosen (hexane, dicholoromethane and methanol) to carry out the experiment because of their positive results for the antimicrobial activity towards the tested microorganism. Apart from leaves, we tested the antimicrobial activity on stems and roots, nevertheless, it did not show any positive activity as leaf extract. Distilled water extracts did not show any antimicrobial activity against *B. subtilis* and *S. aureus.* The same results were obtained by Hassim *et al.*, 2013 [34].

Hexane extract gave the highest activity towards all bacteria tested compared to DCM and methanol (Table 5). The largest inhibition zone (15.5 mm) was produced by hexane extracts against Methilin-resistant *Staphylococcus aureus* (MRSA). The extracts also gave good inhibition zone against *Bacillus cereus*, however, tests against *Enterococcus faecalis* and *Salmonella entiriditis* showed lower activity compared to Ampicillin (control) and other two bacteria.

**Table 5:** Inhibition zone of *P. minus* leaves extract from different solvent extractions.

Sample	Diameter of Inhibition Zone (mm)			
	MRSA	*B. cereus*	*E. faecalis*	*S. entiriditis*
Ampicillin (+control)	17.0	17.5	36.5	37.5
DMSO (−control)	0	0	0	0
Hexane	15.5 ^a^	14.5 ^a^	14.0 ^a^	7.5 ^ab^
DCM	0	13.5 ^a^	0	5.0 ^abc^
Methanol	10.0 ^ab^	12.5 ^a^	11.5 ^b^	0

Means that do not have the same letter within each column differs significantly by the Duncan Multiple Range Test (DMRT) (*p* ≤ 0.05)

Extracts that showed inhibitions zone more than 12.0 mm in diameter were chosen for MIC and MBC determination. The lowest MIC value was 1.25 mg/mL against *B. cereus* (from hexane extracts) and *E. faecalis* (from methanol extracts) (Table 6). The MIC and MBC values (mg/mL) were the same for hexane extract against MRSA (data not shown). When the MIC and MBC were the same values, the drug is considered bacteriostatic. Nevertheless, the MBC value smaller than MIC against *E. faecalis* for methanol extracts in which the effect was bactericidal.

**Table 6:** Minimal Inhibitory Concentration of *P. minus* leaves extracts.

Sample	Concentration (mg·mL^−1^)		
	MRSA	*B. cereus*	*E. faecalis*
Ampicilin	1.0	0.1	0.1
Hexane	5	1.25	5
DCM	n.d	n.d	n.d
Methanol	2.5	2.5	1.25

n.d—not determined.

Although a high concentration is needed to kill the tested bacteria, further investigation is encouraged considering these two factors. First and foremost, samples used in this study were crude extracts, which may not produce better results compared to pure compound, for example, ampicillin, methicillin or chloramphenicol. Second, the extract compounds were able to kill MRSA a bacteria responsible for several infections in humans and which is difficult to treat and may contribute to a new, natural source of antibiotic.

In this study, we also carried out an experiment on the essential oil from different tissues, but no activity was found in the essential oil up to a concentration of 10 mg/mL. It should be highlighted that these results do not mean that there is no antimicrobial activity, as it may be displayed only for higher concentrations, not tested herein.

## 3. Experimental Section

### 3.1. Chemicals

All solvents used were of analytical grade and purchased from Merck (Whitehouse Station, New Jersey, NJ, USA), unless stated. Acetylthiocholine iodide (ATCI), acetylcholinesterase (AChE) type VI-s, from electrical eel, 5,5'-dithiobis [2-nitrobenzoic acid] (DTNB), galanthamine, 1,1-Diphenyl-2-picrylhydrazyl (DPPH), Ascorbic acid, Gallic acid, Follin-Ciocalteu’s reagents, Sodium carbonate, Sodium phosphate, Potassium ferricyanide, Thichloroacetic acid were purchased from Sigma.

### 3.2. Plant Materials

Leaf, stem and root samples from *P. minus* were collected from the INBIOSIS experimental plot on December 2011. Samples were originally collected from Ulu Yam, Malaysia and the voucher specimen was deposited in the UKMB Herbarium, National University of Malaysia. These were identified by taxonomist and further confirmed using ITS sequences [42]*.* Samples washed and stored at −80 °C. For biological activities, the collected plant material (stems, leaves and flowers) was air-dried at ambient temperature. The dried plant material was cut up and stored in paper bags until needed.

### 3.3. Sample Preparation for SPME Technique

Prior to analysis of the volatile compounds, the samples were ground, using a mortar and pestle into a fine powder. The samples were weighted 0.3 g and sealed with a 20 mL headspace glass vial (flat bottom, 100 pk, Perkin Elmer, USA using a PTFE/silicon septum 200 mm. 

### 3.4. Solid Phase Microextraction (SPME) Conditions

The Solid Phase Microextraction (Supelco) method was undertaken using 100 μm PDMS (Polydimethylsiloxane) fibers based on Huang [43]. PDMS fiber was reported to present good sensitivity and high reproducibility among the set of the other fibers [44,45,46]. PDMS fibre was used after optimization of major parameters (time, volume and temperature adsorptions). The fibre and the manual SPME holder were purchased from Supelco (Bellefonte, PA, USA). The fibres were conditioned prior to use, according to the manufacturer’s instructions. About 300 mg of fresh leaves were ground with liquid nitrogen and placed in a 20 mL vial. Optimal conditions of fibre were obtained under the following procedures: 700 uL of distilled water was added to the ground leaves and the vial was covered tightly using a hole cap with septum to ensure no volatile could escape during the extraction. The fibre was then exposed to the sample headspace by inserting the fibre through the septum and the vial with the exposed fibre was incubated in a water bath at 45 °C for 15 min. After 15 min, the fibre was thermally desorbed by inserting the fibre into GC injector at 250 °C for 10 min. Three replicates were used for each analysis.

### 3.5. Isolation of the Essential Oil by Hydrodistillation Technique

300 grams of *P. minus* were subjected to hydrodistillation with 2 L of distilled water for 8 h using a Clevenger-type apparatus to produce a yellowish essential oil. The essential oils were collected over water, separated, dried over nitrogen gas and stored in the dark at 4 °C prior GC-MS and biological activities test. 

### 3.6. Gas Chromatography-Mass Spectrometry Analysis of the Volatile Compounds

The samples were analyzed using a Clarus 600 GC-MS system (Perkin Elmer, Shelton, CT, USA). The compounds were separated using a 30 m × 0.25 mm × 0.25 μm Elite-5MS column (Perkin Elmer, USA). The injector port was heated to 250 °C and the carrier gas was helium at a constant flow of 1 mL·min^−1^. The oven temperature was set at 40 °C for 1 min and then increased at 5 °C·min^−1^ until it reached 250 °C and held for 1 min. All mass spectra were acquired in electron impact mode (EI). The MS parameters were as follows: EI mode, an ionization voltage of 70 eV, an ion source temperature of 200 °C and a scan range of 40–600 Da. The peaks were tentatively identified based on a library search using NIST and Wiley Registry 8 Edition.

### 3.7. GC-FID Analysis and n-Alkane Standard Solutions

In order to perform Kováts indices, samples were analysed using a Perkin Elmer –Clarus 580 system GC-FID. The compounds were separated on 30 m × 0.25 mm × 0.25 μm Elite-5MS column. The GC program was the same as those used for GC-MS analysis. *n*-alkane standard solutions C8-C20 (mixture No. 04070) and C21-C40 (mixture No. 04071) were purchased from Fluka Chemica. Retention indices of essential oil compounds was carried out according to standard method of Kováts Indices to support the identification of the compounds [13].

### 3.8. Data Processing of GC-MS

All peaks exceeding signal to noise ratio (S/N) of 100 were detected. A library search was conducted for peak identification using The National Institute of Standards and Technology (NIST, version 2.0, Gaithersburg, MD, USA) database and all peaks were combined into a single peak table and were then transferred into Microsoft Excel. The volatile information was extracted based on the compounds name and the match and the reverse match value below 800 were filters. After the filtering steps, the new peak tables were formed. The new percentage areas relative to the total percentage area of all compounds were calculated to normalize the data. The above samples were repeated for all samples and all data were combined into a single peak table.

### 3.9. Principal Component Analysis (PCA)

Multivariate stastical analysis of PCA was done as described by Azizan *et al.* 2012 with modifications [47]. Briefly, all the peaks and spectra from each sample were combined into a single peak table and transferred into Microsoft Excel 2007. After a filtering step, the table containing the total counts was imported into SIMCA-P+ 12.0 software (Umetrics, Sweden) for multivariate analysis (PCA). Scaling using the square root of the standard deviation (pareto scaling) was performed during the analysis. The PCA output consisted of score plots in order to visualize the contrast between different samples and loading plots to explain the cluster separation.

### 3.10. Phytochemical Analysis of Plant Extracts

#### 3.10.1. Preparation of Plant Extracts for Biological Activities

Dried, ground plant material was extracted by maceration with water, methanol, ethanol, chloroform, *n*-hexane and dichloromethane. Briefly, 50 g of plant material was soaked with 250 mL of solvent. The plant was macerated three times at room temperature using fresh solvent in every 24 h. The filtrates obtained were combined and then evaporated to dryness using rotary evaporator at 45 °C on a water bath. The aqueous extracts will be dried using freeze drier technique. The obtained extracts were kept in sterile sample tubes and stored at 4 °C.

#### 3.10.2. Determination of Antioxidant Activity

##### 3.10.2.1. Determination of Total Phenolic Content

The total phenolic content was determined using Folin-Ciocalteu’s method [48]. The reaction mixture was prepared by mixing 0.2 mL of methanolic solution of extract (1 mg/mL) and 1.5 mL of 10% Follin-Ciocalteu’s reagent dissolved in water. The mixture was allowed to equilibrate for 5 min and then mixed with 1.5 mL 6% Na_2_CO_3_ solution. After incubation for 90 min at room temperature in darkness, the absorbance of the mixture was read at 725 nm against a blank using spectrophotometer. The blank was prepared with methanol instead of extract solution. The samples were prepared in triplicate and the mean value of absorbance was obtained. The sample procedure was repeated for gallic acid which was used for calibration of standard curve. Total phenol content is reported as gallic acid equivalents by reference to linear equation of the standard curve (y = 0.008x + 0.0077, *R*^2^ = 0.998). Then, the total phenolic content was expressed as milligram of gallic acid equivalent per gram of extract (mg GAE/g of extract).

##### 3.10.2.2. DPPH Radicals Scavenging Capacity Assay

The antioxidant activities of all extracts were evaluated through free radical scavenging effect on 1,1-diphenyl-2-picrylhydrazyl (DPPH) radical. The determination was based on the method proposed by Akowuah *et al.* (2005) [49] with slight modifications. One mL of 0.5 mM DPPH methanolic solution was added into 1 mL of sample extracts (0–2 mg/mL). The mixture was thoroughly mixed and kept in the dark for 1 h. The control was prepared by mixing 1 mL of DPPH and 1 mL methanol. The absorbance was measure at 600 nm using spectrophotometers (Beckmann Counter, Brea, CA, USA). Ascorbic acid and Gallic acid were used as a positive control. Samples were measured in three replicates. Percentage of DPPH scavenging activity was calculated as % inhibition of DPPH = [Abs control − Abs sample/Abs control] × 100. The IC_50_ value is the effective concentration at which 50% of DPPH radicals were scavenged. It was obtained from the graph of scavenging activity (%) *versus* concentration of samples. Low IC_50_ value indicates strong ability of the extract to act as DPPH scavenger. 

##### 3.10.2.3. Ferric Reducing Power Assay (FRAP)

Ferric-reducing antioxidant power was measured by the direct reduction of Fe^3+^(CN^−^)_6_ to Fe^2+^(CN^−^)_6_ and was determined by measuring absorbance resulting from the formation of the Perls Prussian Blue complex following the addition of excess ferric ions (Fe^3+^). Thus, the ferric-reducing antioxidant power (FRAP) method of Oyaizu [50] with slight modifications was used to measure the reducing capacity of samples [51]. This method is based on the reduction of (Fe^3+^) ferricyanide in stoichiometric excess relative to the antioxidants (Gulcin, 2007). Different concentrations of samples 1 mg/mL in 0.75 mL of distilled water were mixed with 1.25 mL of 0.2 M, pH 6.6 sodium phosphate buffer and 1.25 mL of potassium ferricyanide [K_3_Fe(CN)^6^] (1%). The mixture was incubated at 50 °C for 20 min. After 20 min of incubation, the reaction mixture was acidified with 1.25 mL of trichloroacetic acid (10%). Finally, 0.5 mL of FeCl_3_ (0.1%) was added to this solution, and the absorbance was measured at 700 nm. Increased absorbance of the reaction mixture indicates grater reduction capability [52]. The results were expressed as µmol Fe^+2^/g extract. (y = 0.0196x + 0.0830; *r^2^* = 0.9885).

#### 3.10.3. Acetylcholinesterase Inhibitory Activity

##### 3.10.3.1. Buffers

The following buffers were used. Buffer A: 50 mM Tris-HCl, pH 8; buffer B: 50 mM Tris-HCl, pH 8, containing 0.1% bovine serum albumin (BSA); buffer C: 50 mM Tris-HCl, pH 8.0, containing 0.1 M NaCl and 0.02 M MgCl·6H_2_O.

##### 3.10.3.2. Enzyme

Acetylcholinesterase was from electric eel (425 U/mg, 687 mg/protein). Lyophilized enzyme was dissolved in buffer A to make 1000 U/mL stock solution, and further diluted with buffer B to get 0.44 U/mL enzyme for the microplate assay.

##### 3.10.3.3. Acetylcholinesterase Activity Assay

Inhibition of acetylcholinesterase activity was determined using Ellman’s colorimetric method. The reaction mixture consisted of: 25 µL of AChE (0.44 U/mL), 125 µL of 3 mM DTNB in Buffer C, 50 µL of buffer B and 25 µL of sample dissolved in DMSO. The reaction was then initiated by the addition of 25 µL of ATCI. The hydrolysis of acetylcholine was monitored by the formation of yellow 2-nitro-5-sulfidobenzene-carboxylate anion as the result of the reaction of DTNB with thiocholine. The enzymatic hydrolysis of acetylthiocholine was released for 30 min at a wavelength of 405 nm. Galanthamine served as the positive control. Any increase in absorbance due to the spontaneous hydrolysis of the substrate was corrected by subtracting the absorbance before adding the enzyme from the absorbance after adding the enzyme. The percentage inhibition was calculated using the equation:

(1) $$Inhibition\;of\;AChE\;\left(\%\right)=\frac{\Delta A\;Control-\Delta A\;Sample}{\Delta A\;Control}$$

Where Δ*A* control is the absorbance of the control reaction (containing all reagents except the test compound), and Δ*A* sample is the absorbance of test sample. Extract concentration providing 50% inhibition (IC_50_) was obtained by plotting the percentage inhibition against extract concentration.

#### 3.10.4. Determination of Antimicrobial Activity

Antimicrobial activities of the different extracts were first tested using simple screening test. Concentration used for each sample extracts was 10 mg/mL which diluted in 1 mL dimethyl sulfoxide (DMSO). Inoculums containing bacteria in Muller Hinton broth (MHB) were adjusted to 1 × 10^8^ colony forming unit CFU/mL using spectrophotometer at A_620_. It was then swabbed on the MHA surface and left to be dried. This was followed by 10 µL of sample extract onto the swabbed MHA and left incubated at 37 °C for 24–48 h in which Ampicillin was used as a positive drug. Inhibition zone was defined by the diameter of clear zone or zone without bacteria on the agar media. 

The bacteria used for the test were Methicillin-resistant *S. aureus* (MRSA) ATCC 33591, *E. faecalis* ATCC 29212, *B. cereus* ATCC 6464 and *Salmonella* *entiriditis* NCTC 5188. The bacterial strains were grown on Mueller-Hinton agar (MHA) plates at 37 °C for 24 h. 

#### 3.10.5. Determination of Minimal Inhibitory Concentration and Minimal Bactericidal Concentration

The Minimal Inhibitory Concentration (MIC) of the *P. minus* leaves extracts were determined by broth micro dilution method according to the standards of the Clinical and Laboratory Standard Institute [53]. Serial dilutions of extract were made in a sterile 96-well microtiter plate. MHB (100 µL) and bacterial inoculums with concentration of 1 × 10^8^ CFU/mL (100 µL) were also added into each test well then incubated for 24 h at 37 °C. To measure the minimal *P. minus* extracts inhibition, colorimetric assays using 3-(4,5-Dimethylthiazol-2-yl)-2,5-diphenyltetrazolium bromide (MTT) was performed. MTT was added into each test well and incubated at 37 °C for 2 h. MIC was defined as the lowest extract concentration showing yellow colour (no bacteria growth) after the incubation period. 

All the positive results from MIC test were preceded to determine *Minimal Bactericidal* (MBC) test. A wire loop was immersed in clear wells and streaked on MHA. The plates were later incubated at 30 °C for 24–48 h. Positive result of MBC was the concentration that showed no growth of bacteria.

#### 3.10.6. Statistical Analysis

PASW Statistic 18 was used for data analysis. The results are presented as means. Means difference between treatments were examined using Anova, (*p* < 0.05) Tukey Test.

## 4. Conclusions

Here, we present results on the volatile profiling of *P. minus* from different tissues using SPME and hydrodistillation techniques coupled with GC-MS. As one of the essential oil producing crops in Malaysia, volatile profiling of different organs is important to understand the variation of metabolites produced. This could lead to the exploitation of decanal and dodecanal metabolites for example, which were found to be major compounds in the leaves of this plant. Different solvent extracts significantly affected antioxidant, anticholinesetrase and antibacterial activity, and the polar extract ethanol showed highest DPPH radical scavenging activity in both leaf and stem extract. Similarly, with anticholinesterase activity, polar extract (aqueous and methanol) had the highest anticholinesterase activity in leaf extracts. Albeit, in antimicrobial studies, the non-polar leaf extracts (hexane) were the most effective extract for inhibiting MRSA in an antibacterial assay. In this study, essential oil of *P. minus* was found to have antioxidant properties, which are important as an additional value for the flavour and food industry. Further study using bio-guided assay will be carried out in order to isolate the pure compound with promising activity. 
